# Serial Mediation Roles of Alexithymia and Loneliness in the Association Between Family Function and Internet Addiction Among Chinese College Students

**DOI:** 10.3389/fpsyg.2022.874031

**Published:** 2022-05-04

**Authors:** Ying Zhao, Kuo Zhang, Mark D. Griffiths

**Affiliations:** ^1^Mental Health Education Center, Yangzhou University, Yangzhou, China; ^2^Zhou Enlai School of Government, Department of Social Psychology, Nankai University, Tianjin, China; ^3^International Gaming Research Unit, Psychology Division, Nottingham Trent University, Nottingham, United Kingdom

**Keywords:** family function, alexithymia, loneliness, Internet addiction, college students

## Abstract

A lot of previous research has highlighted the negative consequences of Internet addiction. However, relatively few is known about the underlying mechanism for Internet addiction among college students in relation to family function. The present study explored the relationship between family function and Internet addiction among college students, as well as the mediating effects of alexithymia and loneliness. A sample of 783 Chinese college students were administered a number of psychometric scales including the “General Function” subscale of the Chinese version of the Family Assessment Device, Toronto Alexithymia Scale, UCLA Loneliness Scale, and Revised Chinese Internet Addiction Scale. The results showed that family function was negatively associated with Internet addiction; the association was significantly mediated by alexithymia; the association was significantly mediated by loneliness; and alexithymia and loneliness sequentially mediated the association. The total mediating effect was 63.96%. The results of the present study are of great significance to the prevention and intervention of Internet addiction among college students.

## Introduction

Due to the increasing popularity of information technologies, Internet use has become an integral part of individuals’ daily lives. The latest statistics from the China Internet Network Information Center (2021) reported that the number of Internet users in 2021 had exceeded one billion. Internet use is beneficial but in a minority of cases, excessive and/or uncontrolled Internet use may lead to Internet addiction (Li et al., 2021). Internet addiction refers to an individual’s inability to control his or her use of the Internet, resulting in psychological, social, academic, and/or work difficulties (Spada, 2014). Internet addiction has become an increasingly researched topic since the first published paper over 25 years ago (Griffiths, 1996). A growing number of studies have shown that Internet addiction is more prevalent among college and university students than in the general population (e.g., Sharma and Sharma, 2018; Gong et al., 2021) and can have serious negative effects on their physical health (Yang et al., 2019), mental health (Lebni et al., 2020), sleep quality (Wang et al., 2019), and academic performance (Asokan et al., 2019). Previous research has explored the influencing factors of college students’ Internet addiction but most studies have focused on environmental or individual differences separately (e.g., Yang et al., 2016; Tian et al., 2020). There are few studies which have focused on both individual and environmental factors simultaneously, especially the role of personality and emotional factors in the development and maintenance of Internet addiction among college students. Therefore, the present study examined the intrinsic mechanism of college students’ Internet addiction from the perspective of both environment and individual differences.

The family is regarded to being primary among all environmental factors and it has the capability to induce and/or facilitate an individual’s Internet addiction (Li et al., 2014). Family arguably has the greatest influence on children and adolescents and is key in shaping everyday behavior (Naghavi and Redzuan, 2012). However, previous studies on this factor have focused on the one-way interaction of how parents influence their offspring (Gao et al., 2018; Zhang et al., 2019). Many scholars have called for treating the family as a complex system and emphasized the importance of family function (Murphy et al., 2017). Therefore, it is necessary to explore the association between family function and Internet addiction and the psychological mechanisms underlying this association among college students.

### Family Function and Internet Addiction

Family function refers to a model in which the family provides necessary material and spiritual conditions for its members to promote their physical and mental development in a healthy way (Miller et al., 2000). According to family system theory (Miller et al., 2000), the organization and structure of family can influence the behavior pattern of family members. Individuals from dysfunctional families may often adopt some kind of addictive behavior to satisfy their unmet needs in the family (Tafà and Baiocco, 2009). Consistent with this theoretical perspective, empirical studies have shown that good family function is negatively associated with Internet addiction, while poor family function may increase addiction likelihood (Yu and Shek, 2013; Kabasakal, 2015). For example, Yu and Shek (2013) analyzed longitudinal data and found that family function of Hong Kong students significantly predicted their Internet addiction. Likewise, Kabasakal (2015) studied college students and found that poor family function was an important risk factor for Internet addiction. Therefore, the following hypothesis was formulated:

*H1*: Poor family function will be negatively associated with Internet addiction.

### Family Function, Alexithymia, and Internet Addiction

Alexithymia is a multi-dimensional concept, which is related to the emotional difficulties in recognizing, analyzing, and expressing self and others, restricted imagination, and externally oriented thinking styles (Taylor et al., 1997). Increasing evidence has demonstrated that alexithymia is a personality trait (Salminen et al., 2006), and many biological, physiological, and psychological correlates have been reported. As an external factor, family function can affect external behavior by means of internal factors, among which personality is an important internal factor (Zhao et al., 2018). The relationship between family function and alexithymia has been examined. For example, Mazaheri et al. (2014) reported that individuals with good family function had lower levels of alexithymia, while those who reported greater family dysfunction had an increased likelihood of alexithymia (King and Mallinckrodt, 2000).

In addition, individuals with alexithymia often escape the challenges of real life through addictive behaviors (Schimmenti et al., 2017). Alexithymia is often associated with negative emotions (Cevlik et al., 2020). In order to get rid of such emotions, individuals with alexithymia often regard the Internet as an outlet for emotional venting (Heaven et al., 2010). Moreover, individuals with alexithymia have cognitive deficits that prevent them from forming and maintaining friendships with others in real life (Jordan and Smith, 2017). Therefore, these individuals may meet their social needs through the virtual online world (Soliman et al., 2021). Recent empirical research has shown that alexithymia is a predictor of Internet addiction (Lyvers et al., 2016). Scores on Internet addiction screening instruments were significantly higher among individuals with alexithymia than those without alexithymia (Baysan-Arslan et al., 2016). Therefore, the following hypothesis was formulated:

*H2*: Alexithymia will mediate the association between family function and Internet addiction.

### Family Function, Loneliness, and Internet Addiction

Loneliness refers to an unpleasant experience that occurs when an individual’s social network is defective in quantity or quality (Peplau and Perlman, 1982). Loneliness exists as a feeling that can be felt at almost every stage of human life, but is more common among young people, which may be related to developmental tasks in early adulthood (Ummet and Ekşi, 2016). According to the emotional security theory (Davies and Cummings, 1994), when family members face family conflicts, their inner negative emotions are stimulated, aggravating their perceptions and reactions to conflicts and ultimately lead to corresponding problematic behaviors. Therefore, loneliness may play a mediating role in the relationship between family environment factors and individual problem behavior. This view was supported by some empirical studies. For instance, Ma et al. (2020) reported that loneliness plays a mediating role in the relationship between childhood maltreatment and adolescents’ mobile phone addiction. He et al. (2020) reported that loneliness mediated the relationship between parental conflict and adolescents’ addiction to social networking sites. Although the mediating role of loneliness in the relationship between family function and college students’ Internet addiction has not been studied to date, indirect evidence supports the mediating role of loneliness.

Loneliness is a factor that is consistently associated with Internet addiction (Demir and Kutlu, 2016). Tokunaga and Rains (2010) reviewed 94 studies in 22 countries and demonstrated moderate yet consistent associations between loneliness and Internet addiction. In addition, Kim et al. (2009) explored the causal relationship between loneliness and problematic Internet use and found that individuals who suffer from loneliness showed a higher preference for Internet use. Moreover, studies have pointed out that family function can predict individual loneliness level (Maes et al., 2015; Nie et al., 2020). In a collectivist society like China (where the present study was carried out), the family is highly valued. The more frequent contact between family members, the less loneliness they experience (Nie et al., 2020). However, in dysfunctional families, members are estranged from each other and apply ineffective social skills they have learned to their interpersonal communication, leading to rejection by others and enhancing the experience of loneliness (Maes et al., 2015). Therefore, the following hypothesis was formulated:

*H3*: Loneliness will mediate the association between family function and Internet addiction.

### Family Function, Alexithymia, Loneliness, and Internet Addiction

Based on the preceding discussion, alexithymia could mediate the association of family function with Internet addiction independently, and loneliness could mediate the relationship between family function and Internet addiction independently. However, exactly how these two mediators work together remains to be explored. Hayes (2013) posits that when a mediation model contains more than one mediator, these mediators could play a serial-mediating role if they are interrelated. Many empirical studies have shown that alexithymia among individuals is closely related to their feelings of loneliness. For example, a study on 224 undergraduate psychology students found that alexithymia can predict their level of loneliness (Qualter et al., 2009), and Huang and Zhao (2020) noted that college students with high alexithymia have emotional processing defects and cannot establish good interpersonal relationships with others, which can lead to feelings of loneliness. Therefore, the following hypothesis was formulated:

*H4*: Alexithymia and loneliness will serially mediate the association between family function and Internet addiction.

**Figure 1:**
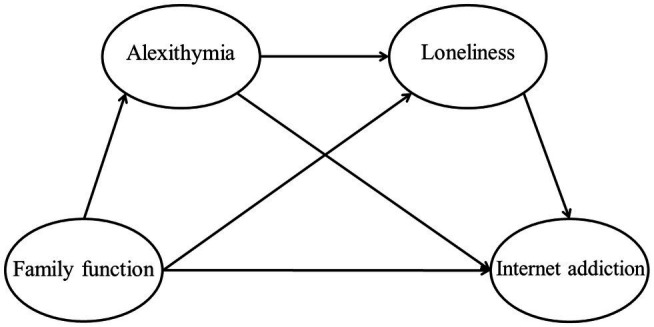
The proposed model.

The proposed model is presented in Figure 1.

## Materials and Methods

### Participants and Procedure

The sample originally comprised 830 college students from two universities in Yangzhou, Jiangsu Province (China). Due to the invalid responses of some participants, a total of 47 surveys were excluded from the analysis. Therefore, the final sample included 783 college students, of which 389 were female (49.7%). The age range of participants was 18–21 years (*M* = 19.52, SD = 1.14).

The study was conducted from August to September 2021. Students in randomly selected classes were invited to participate in the survey. Those enrolling were routed online through *WeChat* (a widely used communication app in China) groups of each class to an informed consent statement. Once they had given consent, they were provided with a link to a web survey (hosted by the Chinese web survey platform *wjx.cn*). All measures were carried out in Mandarin Chinese, and no one received any remuneration. The online survey took approximately 15 minutes to complete. The study was approved by the Research Ethics Committee of the corresponding author’s institution and carried out in accordance with the principles of the Declaration of Helsinki.

### Measures

#### Demographics

Previous studies have shown that participants’ gender, age, and family structure are associated with Internet addiction (Chen et al., 2016; Wu et al., 2016; Malak et al., 2017). Therefore, these three demographic variables were controlled for in the present study. Both gender and family structure were dummy coded (0 = male, 1 = female; 0 = non-intact family, 1 = intact family).

#### Family Function

Family function was assessed using the “General Function” subscale of the Family Assessment Device (FAD; Epstein et al., 1983). The General Function subscale (GF) comprises 12 self-report items (e.g., “We can express our feelings to each other”; “We avoid talking about things we fear and care about”). Items are rated on a four-point scale from *strongly agree* (1) to *strongly disagree* (4). Total scores are calculated after relevant items are reverse coded, with higher scores indicating better family function. The FAD scale has shown good reliability and validity in Chinese participants over 12 years old (Su and Duan, 2008). Cronbach’s alpha coefficient for the GF subscale in the present study was 0.876.

#### Alexithymia

Alexithymia was assessed using the Toronto Alexithymia Scale-20 (TAS-20; Bagby et al., 1994) in the present study. The TAS-20 is a 20-item scale comprising three subscales: difficulty identifying feelings (DIF; e.g., “I have some physical sensations that even a doctor cannot understand”), difficulty describing feelings (DDF; e.g., “People want me to describe more about my feelings”), and externally oriented thinking (EOT; e.g., “I like to talk to others about their daily activities rather than their feelings”). Items are rated a five-point scale ranging from *strongly disagree* (1) to *strongly agree* (5). Total scores are summed after five relevant items are reverse scored, with higher scores representing higher degree of alexithymia. Research shows that TAS-20 is suitable for Chinese people (Zhang et al., 2017). Cronbach’s alpha coefficient for the scale in the present study was 0.870.

#### Loneliness

Loneliness was assessed using the UCLA Loneliness Scale (Russell et al., 1980). The UCLA Loneliness Scale is a 20-item scale (e.g., “I feel no one can ‘trust’: ‘I’ feel no point in interacting with others”) that assesses participants’ feelings of social isolation and loneliness. Items are rated a four-point scale ranging from *never* (1) to *often* (4). Higher scores indicate a stronger sense of social isolation and loneliness. Cronbach’s alpha coefficient for the scale in the present study was 0.914.

#### Internet Addiction

Internet addiction was assessed with the revised Chinese Internet Addiction Scale (CIAS-R; Bai and Fan, 2005). The CIAS-R is a 19-item scale comprising four subscales: compulsive use and withdrawal (e.g., “No matter how tired, I feel very energetic when surfing the Internet”), tolerance (e.g., “I find myself spending more and more time online”), interpersonal and health problems (e.g., “Surfing the Internet has a negative effect on my body”), and time management problems (e.g., “I was told more than once that I spent too much time on the Internet”). Items are rated on a four-point scale ranging from *not at all true* (1) to *always true* (4), with higher scores indicating higher levels of Internet addiction. The scale has been used among Chinese college students and showed good reliability and validity (Tian et al., 2015). Cronbach’s alpha coefficient for the scale in the present study was 0.943.

### Statistical Analysis

The data were analyzed using SPSS 24.0 and Mplus 7.0 (Muthén and Muthén, 2012). First, a descriptive statistical analysis was conducted (e.g., means, standard deviations, skewness and kurtosis values, and correlation analysis). Second, the structural equation modeling (SEM) procedure using the maximum likelihood estimation (ML) method was carried out in Mplus 7.0. Third, the full and partial mediation models were compared. The path from family function to Internet addiction was constrained to zero and then compared with the fit of the model that estimated the path freely. Partial mediation is supported if the fit of two models is significantly different (at least two out of three criteria need to be met: Δ*χ*^2^ significant at, *p* < 0.05, ΔCFI ≤ 0.005, and ΔRMSEA ≤ 0.010; James et al., 2006). Furthermore, a bootstrapping analysis was conducted to examine whether the mediation effect was significant in 1,000 samples, and calculated the 95% confidence interval. The mediating effect is significant when the 95% confidence interval does not contain zero.

The following indicators were adopted to assess the model fit in the present study: the *χ*^2^/df ratio, the Tucker–Lewis Index (TLI), the Comparative Fit Index (CFI), the Root Mean Square Error of Approximation (RMSEA), and Standardized Root Mean Square Residual (SRMR). According to the suggestion of Hu and Bentler (1999), *χ*^2^/df ≤ 3, TLI ≥ 0.95, CFI ≥ 0.95, RMSEA ≤ 0.05, and SRMR ≤ 0.05 are regarded as good model fits; *χ*^2^/*df* ≤ 5, TLI ≥ 0.90, CFI ≥ 0.90, RMSEA ≤ 0.08, and SRMR ≤ 0.08 are regarded as acceptable model fits.

## Results

### Descriptive Analyses

Table 1 presents the means, standard deviations, skewness and kurtosis values and Pearson’s bivariate correlations for all observed variables. As expected, family function was negatively correlated with alexithymia (*r* = −0.43, *p* < 0.001), loneliness (*r* = −0.42, *p* < 0.001), and Internet addiction (*r* = −0.31, *p* < 0.001); alexithymia was positively correlated with loneliness (*r* = 0.52, *p* < 0.001) and Internet addiction (*r* = 0.43, *p* < 0.001); loneliness was positively correlated with Internet addiction (*r* = 0.35, *p* < 0.001). These results provided a foundation for SEM analysis.

**Table 1:** Descriptive statistics and correlations among variables.

S. No.	Variables	*M*	*SD*	Skewness	Kurtosis	1	2	3
1.	Family function	37.61	5.92	−0.37	0.25	1		
2.	Alexithymia	52.47	11.46	0.03	0.18	−0.43^***^	1	
3.	Loneliness	44.69	9.73	−0.25	0.01	−0.42^***^	0.52^***^	1
4.	Internet addiction	45.29	11.16	0.09	0.42	−0.31^***^	0.43^***^	0.35^***^

*N* = 783.

*** *p* < 0.001.

### Measurement Model

The two-step approach suggests that researchers examine the measurement model by confirmatory factor analysis (CFA) before testing the structural relationships (Anderson and Gerbing, 1988). The measurement model of the present study comprised four interrelated latent variables: family function, alexithymia, loneliness, and Internet addiction. Since some variables in the present study are unidimensional, it is more suitable to reduce the risk of convergence problems and improve model fits by using item parceling rather than individual items (Sterba and Rights, 2016). More specifically, according to the item-to-construct balance approach (i.e., allocating the highest and lowest load items in each package in turn), the 12-item FAD-GD and the 20-item UCLA Loneliness Scale were put into two packages, respectively. For the other two scales, TAS-20 and CIAS-R, they were packaged according to their respective dimensions. The results of CFA for the measurement model showed a good fit with the data: *χ*^2^ = 166.644, *χ*^2^/df = 4.385, CFI = 0.973, TLI = 0.961, SRMR = 0.038, and RMSEA = 0.066 (90% CI = 0.056–0.076). All the loadings for the indicators of the latent variables were significant (*p* < 0.001), demonstrating that all the latent constructs were well represented by their indicators.

### Structural Model

SEM was conducted to test the proposed structural relationships among family function, alexithymia, loneliness, and Internet addiction. The results showed that the fit indexes met the requirements: *χ*^2^ = 214.888, *χ*^2^/*df* = 3.160, CFI = 0.970, TLI = 0.961, SRMR = 0.046, and RMSEA = 0.053 (90% CI = 0.045–0.061). Furthermore, tests of parameter estimates showed that all direct path coefficients were significant in the proposed directions. The results showed that alexithymia and loneliness partially mediated the relationship between family function and Internet addiction.

### Full Versus Partial Mediation

**Figure 2:**
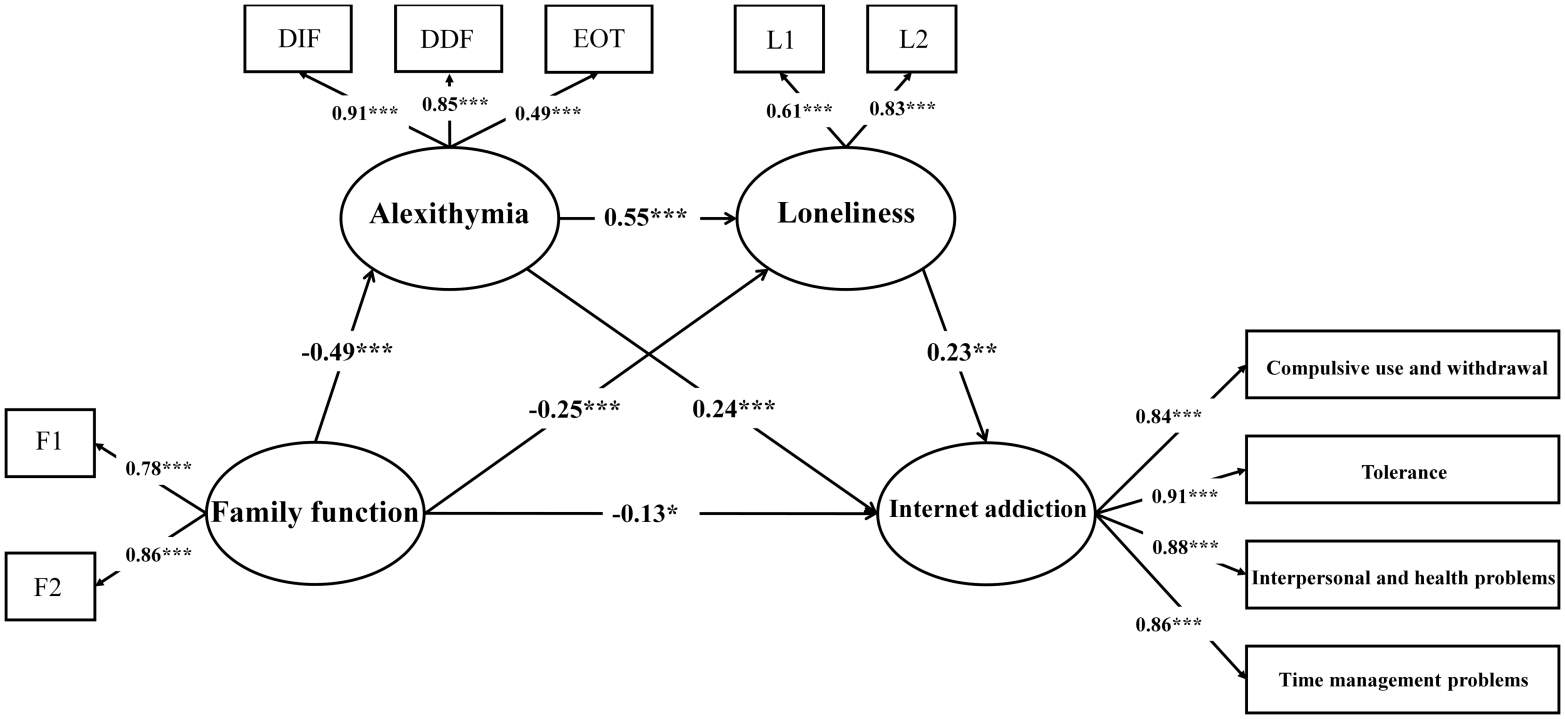
The serial mediation model. All path coefficients are standardized. ^*^p < 0.05, ^**^p < 0.01, ^***^p < 0.001.

To test the assumption that alexithymia and loneliness mediate the relationship between family function and Internet addiction, the following two potential mediation models were compared using a chi-square difference test: (i) a full mediation model with the direct path from family function to Internet addiction constrained to zero; (ii) a partial mediation model with the above direct path not constrained. The chi-square difference test showed that after removing the above direct path, the fit of the model was significantly reduced [Δ*χ*^2^ (1, *N* = 783) = 8.13, *p* < 0.001, ΔCFI = 0.002, and ΔRMSEA = 0.001]. Therefore, the partial mediation model was supported. Path coefficients of the serial mediation model are shown in Figure 2.

### Assessment of Mediation

Total, direct, and indirect effects and their associated 95% confidence intervals (CIs) are shown in Table 2. The results showed that the bootstrap 95% CI of the mediating effect of alexithymia did not include 0 (−0.108, −0.019). The mediating effect was −0.118, accounting for 31.98% of the total effect. Then, the mediating effect of loneliness was tested. Bootstrap 95% CI did not include 0 (−0.123, −0.022). The mediating effect was −0.057, accounting for 15.45% of the total effect. Finally, we examined the mediating effects of alexithymia and loneliness on the influence of family function on Internet addiction and found that the bootstrap 95% CI also excluded 0 (−0.196, −0.056). The mediating effect was −0.061, accounting for 16.53% of the total effect. Therefore, the total mediating effect was 63.96%.

**Table 2:** Direct, indirect and total effects of the pathways tested.

Pathway	Estimate	*SE*	*p*	95% CI	
				Lower	Upper
Direct effect	−0.133	0.058	0.023	−0.245	−0.021
FF → AA→IA	−0.118	0.034	0.001	−0.108	−0.019
FF → LS → IA	−0.057	0.024	0.020	−0.123	−0.022
FF → AA→LS → IA	−0.061	0.024	0.010	−0.196	−0.056
Indirect effect	−0.236	0.040	0.000	−0.327	−0.164
Total effect	−0.369	0.041	0.000	−0.442	−0.281

S, Standard error; FF, Family function; AA, Alexithymia; LS, Loneliness; IA, Internet addiction.

## Discussion

The present study suggests there are four pathways in which family function influences college students’ Internet addiction: direct effect, effect mediated by alexithymia, effect mediated by loneliness, and effect mediated by alexithymia first and then by loneliness second. It is worth noting that approximately 65% of the effect of family function on Internet addiction among college students was realized through the mediating effect. These findings provide a deeper and more nuanced understanding of the internal mechanisms of how family function is associated with Internet addiction, and have practical significance for effective intervention and prevention of Internet addiction. For instance, although a college student’s family function cannot be changed, their cognition can be. In turn, this can change their emotional state, and ultimately have a positive and protective impact on their online behavior.

The research results showed that family function significantly negatively predicted Internet addiction among college students, which is consistent with previous research results (e.g., Yu and Shek, 2013; Kabasakal, 2015) and which supported H_1_. Families can provide emotional warmth and social control for members (Chng et al., 2015). Families with good function have a warm atmosphere and stable social control, and their members have better mental health and less problematic behaviors, such as Internet addiction. Conversely, dysfunctional families may not be able to provide emotional warmth. Family members regard the Internet as a place to provide emotional support resources and are gradually unable to regulate their own use of the Internet (Hou et al., 2017). When coupled with the reduction of social control, members’ behavior patterns are more likely to be inconsistent with their social relationships, increasing the possibility of developing Internet addiction.

The hypothesis that alexithymia would mediate the relationship between family function and Internet addiction (H_2_) was supported. College students with good family function had lower levers of alexithymia and did not use Internet problematically, while those with poor family function had higher levels of alexithymia and were more prone to Internet addiction. Combining the viewpoint of the comprehensive theoretical model of problem behavior (Zhang and Dong, 2011), the external environmental system and individual system contain risk factors and protective factors respectively, and the occurrence of problem behavior has corresponding protective factors and risk factors. The results of the present study suggest that the poor family function of college students increases the level of alexithymia. For college students, family function becomes a risk factor, alexithymia becomes a protective factor, and Internet addiction behavior is the reflection of their self-protection.

The present results also showed that loneliness mediated the relationship between family function and Internet addiction. More specifically, college students with high-quality family function experienced less loneliness and were not dependent on the use of the Internet, while those who reported low-quality family function experienced a stronger sense of loneliness and over-indulged in the online world. The results support H_3_ and extend the findings of previous theoretical and empirical studies. More specifically, the present study applies the emotional security theory to addictive behavior which deepens the understanding of this theory. Although the emotional security theory has been verified by different developmental perspectives, such as internalization and externalization problems (Cummings and Davies, 2011), the present study demonstrates the applicability of the theory in the field of Internet addiction. Likewise, the present study also provides a possible answer to how family function affects Internet addiction among college students. It should be noted that the indirect effect explained by loneliness is not particularly large in the present study. It appears plausible that the effects of bad mood may have long-term effects, which have not been adequately captured in cross-sectional studies. In fact, Davies et al. (2016) found that the proportion of mediation pathways identified in cross-sectional studies was lower than in longitudinal studies.

More importantly, the present study showed that the association between family function and Internet addiction could be sequentially mediated by alexithymia and loneliness, which is consistent with H_4_. Family function not only had a direct impact on Internet addiction, but also had an indirect impact on Internet addiction via alexithymia and loneliness. These results expand existing research on family function, alexithymia, loneliness, and Internet addiction. A good family function not only affects college students’ alexithymia, but also influences the maintenance of intimate relationships which can reduce loneliness (Sharabi et al., 2012) and the possibility of seeking support in the virtual world, and ultimately reducing the incidence of Internet addiction (Yan et al., 2014).

The results complemented the cognitive behavior model of problematic Internet use (Davis, 2001), which proposes that the proximal factor leading to individual problematic Internet use is maladaptive cognition, which in the present study was alexithymia with cognitive deficits in emotion. Distal factors refer to underlying psychopathological factors, such as depression, anxiety, and loneliness in the present study. Cognitive symptoms precede emotional or behavioral symptoms and cause the latter two. Moreover, the individual’s poor social support system appears to be the direct cause of Internet addiction.

## Limitations and Implications

Although the present study explored the underlying mechanisms of how family function affects college students’ Internet addiction, some limitations should also be acknowledged. First, the cross-sectional survey design does not allow us to verify the causal relationship among variables. To solve this limitation, a longitudinal design could be used in future studies. Second, the measures adopted were all self-report. Although there were no serious common methodological biases in the present study, future studies should use mixed research methods to collect data. Third, the sample was self-selecting Chinese students and therefore the findings cannot necessarily be generalized to all Chinese cohorts or other nationalities. To confirm and extend the findings of the present study, future studies should use more nationally representative samples across different countries and cultures.

Despite the limitations of the present study, it has research value and significance. The results of the present study provided some evidence for the relationship between family function and Internet addiction among Chinese college students and enriches the literature in the field of Internet addiction. It also provided an empirical framework by examining the multiple mediating effects of alexithymia and loneliness. The present study combined family and individual factors, while taking personality and emotional state into account, consolidating and broadening the understanding of the potential mechanism between family function and Internet addiction as well as providing multi-perspective educational suggestions for the intervention and prevention of Internet addiction among college students.

## Conclusion and Recommendations

The present study adopted a multiple mediation model to conduct an in-depth study examining the influence of family function on Internet addiction among Chinese college students. The results showed that family function was negatively associated with Internet addiction. Mediating analyses showed that alexithymia and loneliness mediated the relationship between family function and Internet addiction, respectively. Moreover, alexithymia and loneliness sequentially mediated the relationship between family function and Internet addiction among college students.

Given the study’s key findings, the prevention and intervention of Internet addiction among college students requires the efforts and cooperation of families, schools, and society. Parents should create a good family environment, paying close attention to their children’s personality development and emotional state, and work together with schools to supervise their children’s network behavior. Schools should keep abreast of students’ family status, improving their psychological counseling, especially the service system of family counseling, to improve students’ personality development and emotional management ability. Society more generally should continue to invest in professionals in the family education consulting services sector and establish a healthy network environment.

## Data Availability Statement

The raw data supporting the conclusions of this article will be made available by the authors, without undue reservation.

## Ethics Statement

The studies involving human participants were reviewed and approved by the research Ethics Committee in Department of Social Psychology at Nankai University. The patients/participants provided their written informed consent to participate in this study.

## Author Contributions

YZ: conceptualization, investigation, data analysis, and writing original draft. KZ: conceptualization, supervision, review, and editing. MG: interpretation, review, writing, and editing. All authors contributed to the article and approved the submitted version.

## Funding

This research was supported by Tianjin Philosophy and Social Science Research Grant (No. TJJX19-007).

## Conflict of Interest

The authors declare that the research was conducted in the absence of any commercial or financial relationships that could be construed as a potential conflict of interest.

## Publisher’s Note

All claims expressed in this article are solely those of the authors and do not necessarily represent those of their affiliated organizations, or those of the publisher, the editors and the reviewers. Any product that may be evaluated in this article, or claim that may be made by its manufacturer, is not guaranteed or endorsed by the publisher.
